# 

**Published:** 1855-10

**Authors:** 


### CONTENTS.

### *ORIGINAL COMMUNICATIONS.*

PAGE.

ARTICLE I.—Pathology of Fevers. By J. G. WESTMORELAND, M. D., Professor of Materia Medica and Medical Jurisprudence, in the Atlanta Medical College........ 65

ARTICLE II.—Secretion of Milk, excited by Nursing, in the Case of a Grandmother. By J. BORING, M. D., Professor of Obstetrics and Diseases of Women and Children in the Atlanta Medical College........ 68

ARTICLE III.—On Chronic Organic Liver Affections. By A. W. GRIGGS, M. D., Newnan, Georgia........ 70

ARTICLE IV.—Obstinate Vomiting in Pregnancy, with Abortion. By Jos. P. LOGAN, M. D., Professor of Physiology and General Pathology in the Atlanta Medical College........ 75

ARTICLE V.—The Medical Virtues of the Compound Fluid Extract of Tephrosia Virginiana. By B. O. JONES, M. D., Atlanta, Georgia........ 77

ARTICLE VI.—New Chemical and Philosophical Apparatus. By ALEXANDER MEANS, M. D., Oxford, Georgia........ 80

ARTICLE VII.—Report of the Surgical Clinic of the Atlanta Medical College. By W. F. WESTMORELAND, M. D., Professor of the Principles and Practice of Surgery........ 86

### *SELECTIONS.*

Food and its Adulterations........ 89

Inoculation in Yellow Fever........ 97

Uterine Pains and Hemorrhage after Delivery........ 103

Lectures on Clinical Surgery........ 109

Alleged Cure of Cancer........ 116

Note on the Induction of Sleep and Anæsthesia by Compression of the Carotids........ 117

A New Operation for Lacerated Perinæum........ 118

Editorial and Miscellaneous........ 119

